# FGG-NUFFT-Based Method for Near-Field 3-D Imaging Using Millimeter Waves

**DOI:** 10.3390/s16091525

**Published:** 2016-09-19

**Authors:** Yingzhi Kan, Yongfeng Zhu, Liang Tang, Qiang Fu, Hucheng Pei

**Affiliations:** 1 College of Electronic Science and Engineering, National University of Defense Technology, Changsha 410073, China; kanyingzhi@nudt.edu.cn (Y.K.); tangliangcool@163.com (L.T.); fuqiang1962@vip.sina.com (Q.F.); 2 Beijing Institute of Mechanical and Electrical Engineering, Beijing 100074, China; peihch@163.com

**Keywords:** near-field, millimeter wave, 3-D imaging, FGG-NUFFT

## Abstract

In this paper, to deal with the concealed target detection problem, an accurate and efficient algorithm for near-field millimeter wave three-dimensional (3-D) imaging is proposed that uses a two-dimensional (2-D) plane antenna array. First, a two-dimensional fast Fourier transform (FFT) is performed on the scattered data along the antenna array plane. Then, a phase shift is performed to compensate for the spherical wave effect. Finally, fast Gaussian gridding based nonuniform FFT (FGG-NUFFT) combined with 2-D inverse FFT (IFFT) is performed on the nonuniform 3-D spatial spectrum in the frequency wavenumber domain to achieve 3-D imaging. The conventional method for near-field 3-D imaging uses Stolt interpolation to obtain uniform spatial spectrum samples and performs 3-D IFFT to reconstruct a 3-D image. Compared with the conventional method, our FGG-NUFFT based method is comparable in both efficiency and accuracy in the full sampled case and can obtain more accurate images with less clutter and fewer noisy artifacts in the down-sampled case, which are good properties for practical applications. Both simulation and experimental results demonstrate that the FGG-NUFFT-based near-field 3-D imaging algorithm can have better imaging performance than the conventional method for down-sampled measurements.

## 1. Introduction

In recent years, the demand for millimeter wave imaging techniques has increased in the field of nondestructive testing (NDT) or in security check applications since the threat of terrorism attacks is increasing [1,2,3,4,5]. Millimeter waves are an effective sensing method since these waves have the ability to penetrate clothing and other dielectric barriers with minimal reflection and attenuation. In addition, three-dimensional (3-D) images can provide more information than two-dimensional (2-D) images, which makes the millimeter wave 3-D imaging promising. Various techniques have been proposed to exploit the abilities of millimeter wave 3-D imaging, such as optimized-based microwave imaging [6], confocal radar-based imaging [7], the microwave tomography method [8], and the microwave holography method [9].

The near-field 3-D images are obtained by illuminating the object at multiple angles using a wideband electromagnetic wave with a spherical wavefront and measuring the scattered fields. The measurements can be achieved with a cylindrical synthetic aperture or 2-D planar synthetic aperture [10]. In this paper, we discuss the mode of 2-D planar synthetic aperture, as it is easy to acquire the scattered data and is the basic model for other types of 3-D imaging systems. The 3-D imaging with wideband 2-D planar antenna aperture can be considered holographic radar imaging with a wideband signal, and can also be regarded as an extension of 2-D SAR imaging to 3-D by adding an additional scanning axis. The 3-D resolutions depend mostly on the aperture of the 2-D plane array and the bandwidth of transmitted radar signal [11].

It is usually difficult to get satisfactory imaging accuracies using the plane wave illumination for high frequencies or large objects in the near-field case [12]. Based on spherical wave propagation theory, many approaches have been proposed to deal with the near-field imaging. The near-field tomographic algorithm could be used to reconstruct an image in Cartesian coordinates [13]. The spherical back-projection method [14] and the fast cyclical convolution method [15] could also be applied to near-field imaging. These algorithms use summation procedures and are more time-consuming than fast Fourier transform (FFT) procedures.

As a matter of fact, the near-field 3-D spatial spectrum lies on a spherical curve and is nonuniform in the 3-D Cartesian domain, which limits the direct application of Fourier transforms. Conventional near-field 3-D holographic imaging methods [1,3] generally use 3-D interpolation to obtain uniform spatial spectrum samples and then apply a 3-D fast Fourier transform to obtain the 3-D image of the target. These approaches can be considered interpolation-FFT methods, which would introduce interpolation errors and have a high demand for densely sampled data especially when the working frequency is high and the imaged scene is small, where the interpolation errors will be serious and greatly affect the quality of reconstructed images. To solve this nonuniform Fourier transforming problem and to take advantage of FFT, nonuniform fast Fourier transform (NUFFT) approaches have been developed. The NUFFT idea was first described by Dutt and Rokhlin [16] and Beylkin [17]. They presented a group of algorithms to generalize the fast Fourier transform in the case of noninteger frequencies and nonequispaced nodes based on a combination of certain analytical considerations with the classical fast Fourier transform. Later, Liu [18] and Nguyen [19] proposed an accurate algorithm for NUFFT based on the regular Fourier matrix, which is a new class of matrices. Then, Greengard [20] improved the computational efficiency of nonuniform Fourier processing based on a fast Gaussian gridding (FGG) scheme by utilizing the nice property of the Gaussian spreading function. The NUFFT approaches have broad applications. For example, they have been used in near-field 2-D imaging to speed up the fast gridding algorithm [12] and can be applied to 2-D and 3-D MR image reconstruction [21,22]. The NUFFT reconstruction method has a better trade-off between accuracy and efficiency than the conventional gridding method and has been successfully applied to studies on small animals [22]. The NUFFT method has also been used in three-dimensional cases to deal with landmine detection using ground-penetrating radar [23]. Compared with the conventional migration method, the NUFFT migration method is more useful in focusing images, estimating landmine structures, and retaining a relatively high signal-to-noise ratio. In [24], to realize linear MIMO array imaging, the authors utilized NUFFT to replace Stolt interpolation, which leads to improvement of the accuracy and computational efficiency.

In this paper, we investigate the effectiveness of the FGG-based NUFFT (FGG-NUFFT) method in near-field millimeter wave 3-D imaging and propose a FGG-NUFFT-based 3-D imaging method. For near-field 3-D imaging, the nonuniform Fourier transforming problem directly affects the imaging quality. According to the nice feature of the fast Gaussian gridding method described in [20], we introduce the fast Gaussian gridding based NUFFT method to solve the nonuniform Fourier transforming problem. The imaging performance of our proposed method in both adequate sampling rate and down-sampling rate are analyzed and validated. Moreover, our method can preserve the FFT’s advantage of high efficiency, making it an available method used for near-field millimeter wave 3-D imaging application in a down-sampled or sparsely sampled situation.

The rest of this paper is organized as follows. In Section 2, a near-field 3-D imaging scene with a two-dimensional plane antenna array is described. Section 3 briefly describes the conventional holographic 3-D imaging algorithm. Section 4 presents our FGG-NUFFT-based 3-D imaging method in detail. Section 5 displays both simulation and experimental results. Finally we summarize the conclusions in Section 6.

## 2. Near-Field 3-D Imaging Scene Description

**Figure 1:**
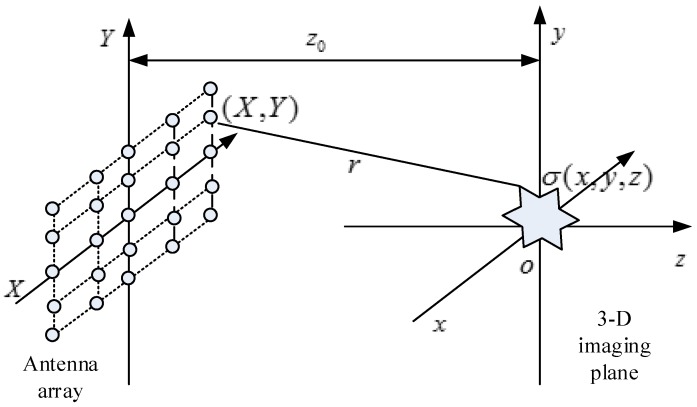
Geometry of near-field 3-D imaging by 2-D plane array.

The monostatic two-dimensional plane antenna array layout for near-field 3-D imaging is shown in Figure 1. The capital coordinates represent the position of the transceiver, and the ordinary coordinates represent a single point in the target or the imaging region. Scattered data are collected by scanning a transceiver over a rectilinear planar aperture that has one or more targets within its field of view. The dimension perpendicular to the 2-D X−Y plane is defined as the *Z*-dimension and the measuring plane is at $Z=z_{0}$. The target is characterized by a reflectivity function σ(x,y,z). Since the probe is within the near-field of the specimen under test, the wavefront curve is no longer negligible. The received spherical wavefront at position (X,Y), with a temporal wavenumber k, is then given by

(1) E(X,Y,k)=∭σ(x,y,z)⋅exp(−jk⋅r)dxdydz

where

(2) $$\begin{array}{l} r=\sqrt{{\left(x-X\right)}^{2}+{\left(y-Y\right)}^{2}+{\left(z-z_{0}\right)}^{2}}\\ k=2\pi f/c=\sqrt{k_{x}^{2}+k_{y}^{2}+k_{z}^{2}} \end{array}$$

Here, r is the range between the probe and the target, c is the speed of light, f is the temporal frequency, $k_{x}$, $k_{y}$ are wavenumbers corresponding to x and y, and the wavenumber corresponding to z is $k_{z}=\sqrt{k^{2}-k_{x}^{2}-k_{y}^{2}}$.

## 3. Conventional Holographic 3-D Imaging

The conventional holographic method is processed in wavenumber domain and its major advantage is high computational efficiency. Based on the theory that the spherical wave can be decomposed into an infinite superposition of plane waves [1], the echoed data can be presented as:

(3) $$\begin{array}{c} E(X,Y,k)=∭\sigma (x,y,z)\cdot \exp(-jk\cdot r)dxdydz\\ =\iint \{∭\sigma (x,y,z)\exp[-j(xk_{x}+yk_{y}+zk_{z})]dxdydz\\ \cdot \exp(jz_{0}k_{z})\exp[j(Xk_{x}+Yk_{y})]\}dk_{x}dk_{y} \end{array}$$

Let $\sigma^{F}(k_{x},k_{y},k_{z})=FT_{3D}[\sigma (x,y,z)]$, we have

(4) $$E(X,Y,k)=IFT_{2D}[\sigma^{F}(k_{x},k_{y},k_{z})\exp(jz_{0}\sqrt{k^{2}-k_{x}^{2}-k_{y}^{2}})]$$

Since the coordinate systems coincide after the FFT and inverse FFT (IFFT) operations, we can ignore the capital and ordinary coordinate systems’ difference and solve Equation (4). Then the 3-D image is reconstructed by [1]

(5) $$\sigma (x,y,z)=IFT_{3D}\{FT_{2D}[E(X,Y,k)]\exp(-jz_{0}\sqrt{k^{2}-k_{x}^{2}-k_{y}^{2}})\}$$

Here, $IFT_{3D}$ indicates a 3-D spatial inverse Fourier transform from the wavenumber domain variables $\left(k_{x},k_{y},k_{z}\right)$ to the spatial domain variables (x,y,z). $FT_{2D}$ indicates a 2-D spatial Fourier transform from the spatial domain variables (X,Y) to the wavenumber domain variables $\left(k_{x},k_{y}\right)$. $FT_{3D}$ and $IFT_{2D}$ are the corresponding inverse transforms of $IFT_{3D}$ and $FT_{2D}$ respectively.

Since the frequencies are uniformly sampled and the probe scans along the X−Y plane with uniform steps, k, $k_{x}$, $k_{y}$ are uniform, making $k_{z}$ nonuniform. Thus Equation (5) cannot be directly solved using the regular FFT algorithm. The conventional imaging methods [1,3] utilize Stolt interpolation to solve this nonuniform Fourier transforming problem, which can be regarded as interpolation-FFT methods. Several methods of realizing Stolt interpolation have been described in [25]. Since the 3-D spatial spectrum here is only nonuniform along $k_{z}$, it is easy for us to use a linear interpolator along $k_{z}$ to realize interpolation and obtain a uniform 3-D spatial spectrum. The major advantage of the interpolation-FFT methods is the high imaging efficiency from utilizing FFT. However, the interpolation procedures introduce approximation errors and the interpolation accuracy depends mostly on the nonuniform spatial spectrum samples. If the measurements are down-sampled or some samples are missed, the imaging quality with the interpolation-FFT methods would be degraded.

## 4. FGG-NUFFT-Based 3-D Imaging

In this paper, a FGG-NUFFT-based 3-D imaging algorithm is introduced to efficiently and accurately deal with the nonuniform summation problem in near-field image reconstruction. The NUFFT approaches are popular methods to overcome the nonuniform sampling limitation [16]. The FGG-NUFFT means fast Gaussian gridding based NUFFT, which was proposed in [20] to accelerate the nonuniform Fourier transforms. A nonuniform Fourier transform is done along $k_{z}$. The process of calculating nonuniform inverse Fourier transform along $k_{z}$ can be presented as 1-D type-1 NUIFFT, where type-1 means a Fourier transform from a nonuniform domain to a uniform domain.

For simplicity, we now consider a general 1-D type-I NUIDFT, which is defined in the form of

(6) $$F(u)=\frac{1}{N}\sum_{i=0}^{N-1}f_{i}e^{-juv_{i}}\;for\;u=-\frac{M}{2},...,\frac{M}{2}-1$$

where $v_{i}\in [0,2\pi ]$ and $v_{i}$ may not be uniform. $f_{i},i=0,\cdots ,N-1$ is nonuniform spectrum data in wavenumber domain, and F(u) is the uniform output signal in spatial domain.

Let f(v) be a periodic function on [0,2π] with $f(v)=\sum_{i=0}^{N-1}f_{i}\delta (v-v_{i})$ and $g_{\tau }(v)$ be the periodic heat kernel on [0,2π] with $g_{\tau }(v)=\sum_{l=-\infty }^{\infty }e^{-{\left(v-2l\pi \right)}^{2}/4\pi }$. $f_{\tau }(v)$ is defined as the convolution with $f_{\tau }(v)=f*g_{\tau }(v)=\int_{0}^{2\pi }f(s)g_{\tau }(v-s)ds$. Then $f_{\tau }(v)$ is a 2π periodic $C^{\infty }$ function, which is well-resolved by a uniform mesh in v, whose spacing is determined by τ. Set the oversampling number $M_{r}=R\times N$, where R is the oversampling coefficient. If we choose grids with a proper interval determined by $M_{r}$, as described in [20], we have:

(7) $$F_{\tau }(u)\approx \frac{1}{M_{r}}\sum_{m=0}^{M_{r}-1}f_{\tau }(2\pi m/M_{r})e^{-ju2\pi m/M_{r}}$$

where

(8) $$f_{\tau }(2\pi m/M_{r})=\sum_{i=0}^{N-1}f_{i}g_{\tau }(2\pi m/M_{r}-x_{i})=\sum_{i=0}^{N-1}f_{i}\sum_{l=-\infty }^{\infty }e^{-{\left(v_{i}-2\pi m/M_{r}-2l\pi \right)}^{2}/4\tau }$$

Once the values $F_{\tau }(u)$ are known, we can get the evaluation of F(u): $F(u)=\sqrt{\frac{\pi }{\tau }}e^{u^{2}\tau }F_{\tau }(u)$. This is a direct result of the convolution theorem and the fact that the Fourier transform of $g_{\tau }$ is $G_{\tau }(u)=\sqrt{2\tau }e^{-u^{2}\tau }$.

The dominant task in the NUFFT is to calculate $f_{\tau }(2\pi m/M_{r})$ in Equation (8), referred to as the gridding process. In fact, the calculation of Equation (8) is actually not that expensive. As the Gaussian heat kernel is sharply peaked, we can change our point of view from the receiving point $2\pi m/M_{r}$ to the source point $v_{i}$ and consider one Gaussian source at a time; then we have

(9) $$f_{\tau }(m+m^{\prime })\leftarrow f_{\tau }(m+m^{\prime })+f_{i}e^{-{\left(v_{i}-\xi -2\pi m^{\prime }/M_{r}\right)}^{2}/4\tau }$$

where $2\pi m/M_{r}$ is the nearest regular grid point from $v_{i}$, $m^{\prime }=-M_{sp}+1,...,M_{sp}$, and $M_{sp}$ is the spreading number. This processing can accelerate the convolution calculation. Details of this algorithm can be found in [20]. The procedure of FGG-NUIFFT processing is described in Table 1. Then, the image reconstruction procedure can be represented as

(10) $$\sigma (x,y,z)=IFT_{k_{x},k_{y}}\{NUIFFT_{k_{z}}\{FT_{2D}[E(X,Y,k)]\exp(-jz_{0}\sqrt{k^{2}-k_{x}^{2}-k_{y}^{2}})\}\}$$

where $NUIFFT_{k_{z}}$ indicates a NUIFFT from $k_{z}$ to z, and $IFT_{k_{x},k_{y}}$ indicates a 2-D spatial inverse Fourier transform from $\left(k_{x},k_{y}\right)$ to (x,y).

## 5. Experimental Results and Analysis

In this section, the performance of our proposed FGG-NUFFT based near-field 3-D imaging method is demonstrated using the simulated and real measured data. The conventional imaging method is utilized for comparison. Both simulated data and real measured data are used to analyze the performance. In the interpolation-FFT method, first-order linear interpolation is employed as it is commonly used in practice [23]. For the calculation of NUFFT, the kernel size $M_{sp}$ and oversampling rate R are set to 4 and 2, respectively. We discuss the FGG-NUFFT based 3-D imaging method in the case of sufficient sampling rate and down-sampling rate, respectively. The latter is meaningful for reducing measuring time and memory.

### 5.1. Simulated Data Imaging

#### 5.1.1. Imaging with Sufficient Sampling Rate

The simulated scattered data are generated by Matlab on a computer, according to the near-field modeling parameters we set. In this simulation case, we assume that the transceiver antenna transmits a wideband signal whose frequency range is f=30~40 GHz with a frequency interval of 100 MHz. It scans along a planar array of 15 cm × 15 cm with a sampling interval of $\Delta x=\Delta y=\lambda_{\min}/4=1.875\;\mathrm{mm}$, where $\lambda_{\min}$ is the minimum wavelength of the wideband signal. The sampling rate in the spatial domain is subject to the rigorous Nyquist sampling rate as described in [1]. The whole area to be imaged is 20 cm × 20 cm × 20 cm. There are seven ideal point-like scatterers with unit reflectivity amplitude located in the imaged area, and the perpendicular distance from the center of the imaged area to the antenna plane is 0.32 m. The coordinates of the scatterers are shown in Table 2. Then a matrix of size 161 × 161 × 101 is generated to store the simulated scattered data of these ideal point-like scatterers, according to Equation (1).

**Figure 2:**
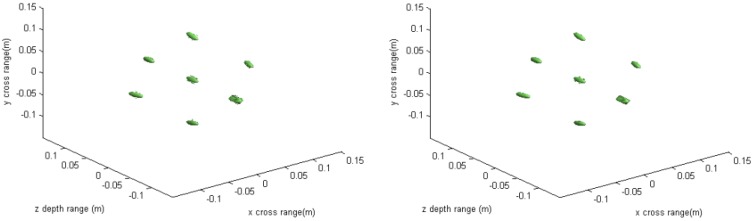
3-D imaging result of the conventional method (**left**) and the proposed method (**right**).

**Figure 3:**
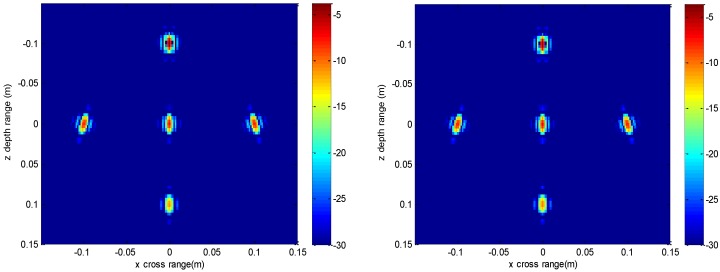
Slice result at *y* = 0 m of the conventional method (**left**) and the proposed method (**right**).

**Figure 4:**
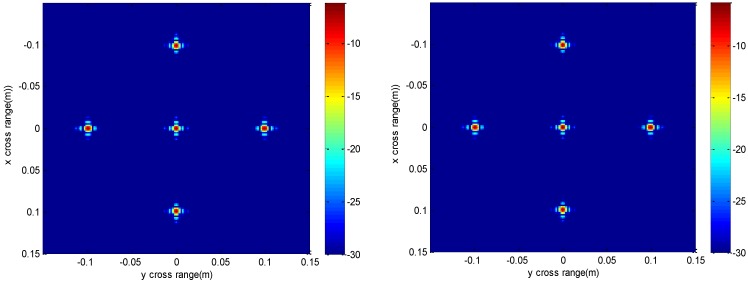
Slice result at *z* = 0 m of the conventional method (**left**) and the proposed method (**right**).

**Figure 5:**
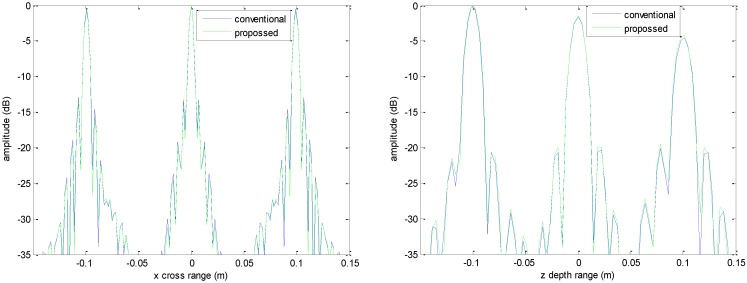
Profile comparisons along *x* direction (**left**) and *z* direction (**right**).

The imaging results depicted in Figure 2, Figure 3, Figure 4 and Figure 5 are acquired by the conventional method and our proposed method with ideal conditions, where noise is absent and the number of samples is adequate. Figure 2 is the reconstructed 3-D image with a dynamic range of −30~0 dB. Figure 3 is the slice at *y* = 0 m, Figure 4 is the slice at *z* = 0 m, and Figure 5 shows the profiles along x and z of the center point in the imaged area. It should be noted that the profile along y is the same with x and can be omitted here. We can see that the point-like scatterers are well focused at the correct locations with two methods and the imaging resolutions are nearly the same. Imaging processes are implemented using Matlab 2012b on a desktop with an Intel Core i5 3 GHz CPU and 16 GB RAM. The computational time for the conventional method with 256 × 256 × 256 FFT points in this simulation is 58.2284 s, while for our proposed method it is 70.3315 s, which is comparable to the conventional method. The conventional interpolation method is accurate and efficient when the sampled data are dense enough. When the samples are not enough, such as in the case of down-sampling, the conventional method does not perform so well, which will be discussed in the next section.

#### 5.1.2. Imaging with Down-Sampling Rate

To further compare the performance of the proposed method and the conventional method, the imaging case with down-sampling rate is discussed. If the scattered data are down-sampled measured with a factor of 4 along both X and Y directions, the measuring time can be reduced to 1/16 of the original time, which is beneficial to data collection.

**Figure 6:**
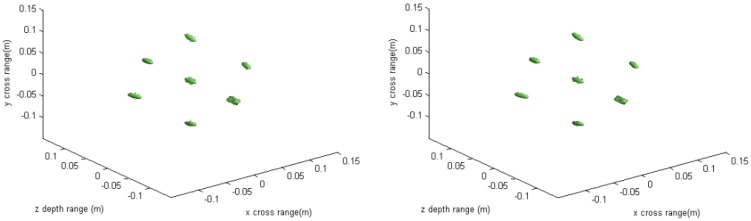
3-D imaging result under down-sampling rate of the conventional method (**left**) and the proposed method (**right**).

**Figure 7:**
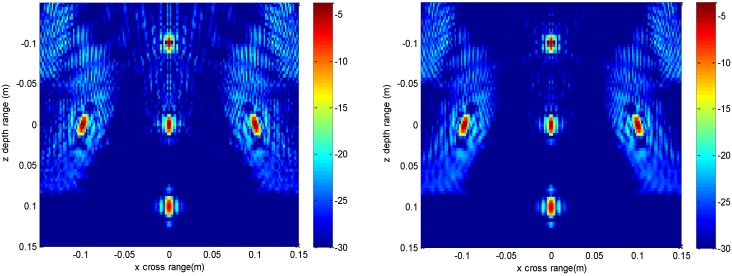
Slice result at *y* = 0 m under down-sampling rate of the conventional method (**left**) and the proposed method (**right**).

**Figure 8:**
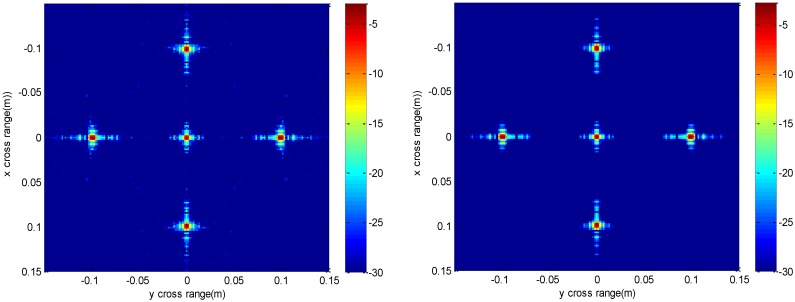
Slice result at *z* = 0 m under down-sampling rate of the conventional method (**left**) and the proposed method (**right**).

**Figure 9:**
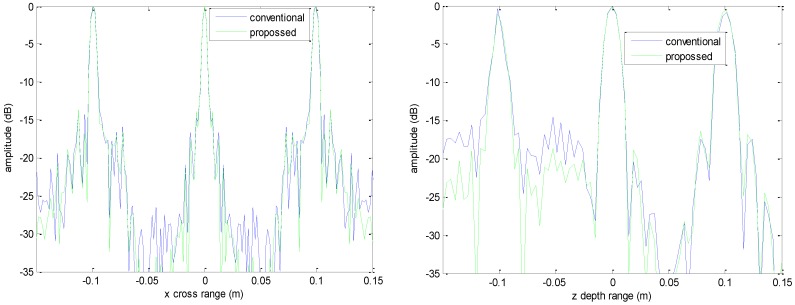
Profile comparisons under down-sampling rate along *x* direction (**left**) and *z* direction (**right**).

The imaging results with a down-sampling rate of 4 using two methods are shown in Figure 6, Figure 7, Figure 8 and Figure 9. Figure 6 is the reconstructed 3-D image, where a dynamic range of −20~0 dB is used to provide a good view. Figure 7 is the slice at *y* = 0 m with a dynamic range of −30~0 dB, where the conventional method has visibly stronger clutter compared to the proposed method. Furthermore, it can be seen that for both methods, there is clutter distributed around the imaged scatterers, and the clutter is heavier when the *z*-coordinates are smaller. Here, we will provide some explanation. For targets located far away from the antenna array, which means the *z*-coordinates are small, the practical spatial spectrum contributed by the targets will be more uniform than that of the targets whose *z*-coordinates are larger. This means the nonuniform Fourier transforming procedure of those targets with smaller *z*-coordinates will introduce fewer errors and have better imaging results. Figure 8 is the slice at *z* = 0 m where it can be seen that compared to the conventional method, our method has relatively weaker sidelobes. Moreover, the slice of the conventional method has many tiny noisy artifacts distributed randomly around the imaged target, while our method has a much better view. Figure 9 shows the profiles along *x* and *z* of the center point in the imaged area, and we can see that our method is more consistent with weaker sidelobes than the conventional method. When looking closely at the profile comparisons along *z*, it can be seen that our method has a slightly higher sidelobe level than the conventional method when it is closer to the main lobe. This can possibly be explained as follows: each interpolation along $k_{z}$ for $\left(k_{x}=0,\;\;k_{y}=0\right)$ is accurate since the spatial spectrum along this line of $k_{z}$ is uniform, while the FGG-NUFFT will still introduce errors for its convolution procedure.

It is demonstrated that the proposed method with down-sampling rate of factor 4 can maintain the imaging performance with sufficient sampled data. Furthermore, the proposed method is better focused with lower sidelobes and weaker clutters than the conventional method in the case of down-sampled data. Computational time of the conventional method with 256 × 256 × 256 FFT points in this simulation is 57.89 s, while for our proposed method it is 75.25 s, which is comparable to the conventional method. It can be seen that the interpolation-FFT method is always fast and efficient, while our method takes more time. The theoretical analysis has been described in Section 4. The main advantage for down-sampling case is to save the processing time for measuring.

### 5.2. Real Data Imaging

The experimental dataset was also used to validate the proposed method in a down-sampling case. In this experiment, the parameters are the same as those in the simulation case with a down-sampling rate of factor 4. There is a pair of metal scissors located with a perpendicular range of 0.32 m to the measured antenna array. The scissors are concealed tightly in a paper box and the millimeter wave can penetrate the thick paper to image the scissors. The scissors are nearly 10 cm×8 cm×1 cm (x−y−z) in size. The measured scattered data are collected by the near-field millimeter wave imaging experimental system which mainly consists of a vector network analyzer (VNA), millimeter wave antennas and a 2-D plane mechanical scanner with millimeter stepped interval. An Agilent E8363A is used as for power and a pair of standard gain horn antennas is linked to the VNA to transmit the millimeter wave signal and receive scattered data respectively. The frequency range is 30~40 GHz with 101 frequency steps. The two antennas are located next to each other to ensure that the imaging mode can be approximately regarded as monostatic. The antennas move on the 2-D plane mechanical scanner step by step with an interval of $\Delta x=\Delta y=\lambda_{\min}=7.5\;\mathrm{mm}$ which is under-sampled with a factor of 4 compared to the rigorous sampling rate. The measuring scope is (−0.15 m,0.15 m)×(−0.15 m,0.15 m) in X−Y plane, and 81 × 81 steps are needed. The average measuring time along X direction is 90 s, so that the whole data acquisition procedure needs 90×81 s=121.5 min. Although this measuring time is long, compared with the procedure under the rigorous full sampling rate, the down-sampled procedure can save 15/16 of time.

**Figure 10:**
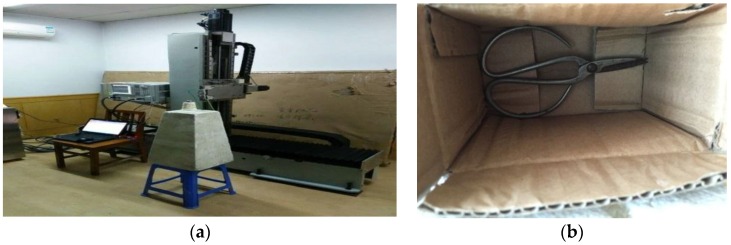
Experimental imaging scene. (**a**) Experimental imaging system; (**b**) Scissors to be imaged.

The experimental imaging system is shown in Figure 10a and the target scene to be imaged is shown in Figure 10b. The paper box will be put on the white foam pillar.

**Figure 11:**
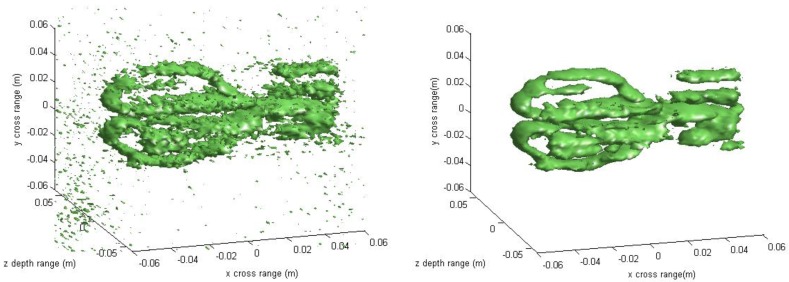
3-D reconstructed result of real data under down-sampled rate using the conventional method (**left**) and the proposed method (**right**).

**Figure 12:**
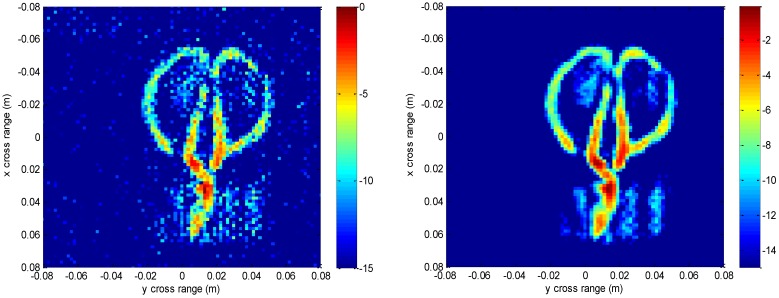
Slice result at *z* = 0 m of real data under down-sampled rate using the conventional method (**left**) and the proposed method (**right**).

**Figure 13:**
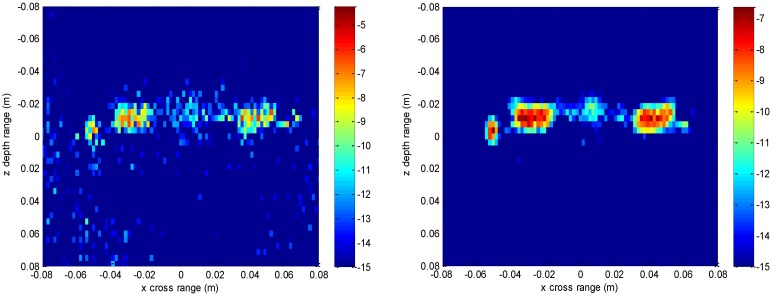
Slice result at *y* = 0 m of real data under down-sampled rate using the conventional method (**left**) and the proposed method (**right**).

**Figure 14:**
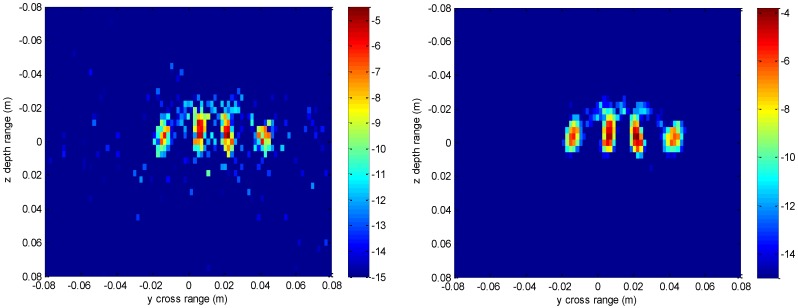
Slice result at *x* = 0 m of real data under down-sampled rate using the conventional method (**left**) and the proposed method (**right**).

**Figure 15:**
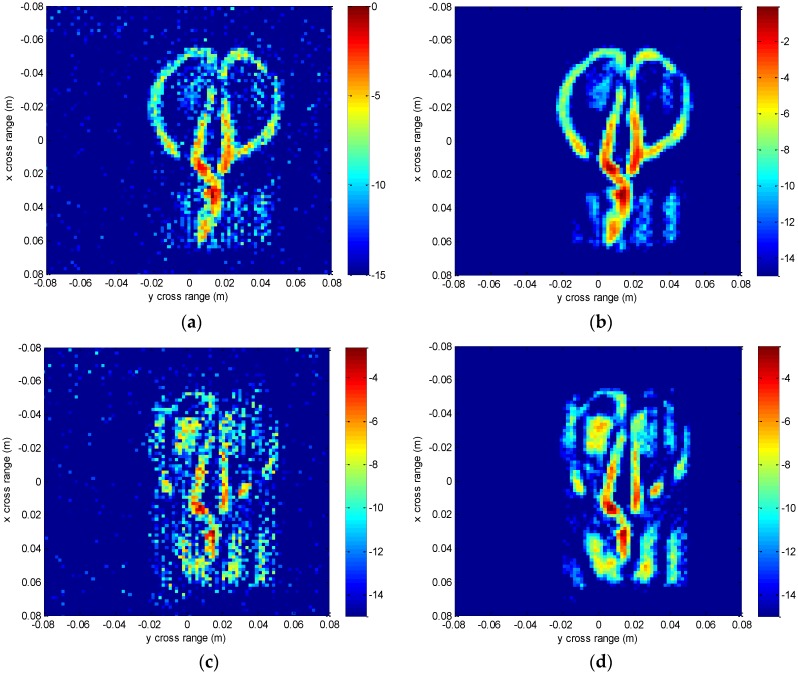

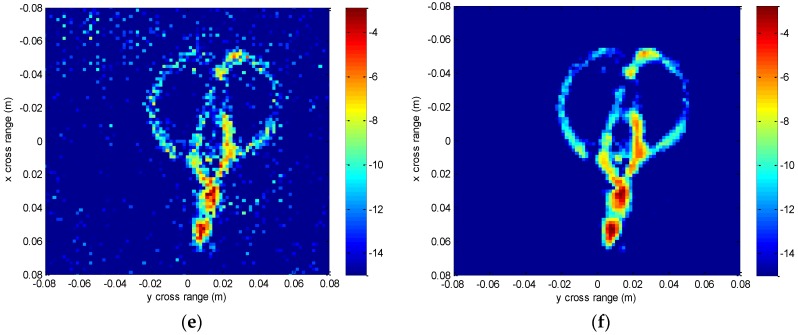
Slices at different *z* depths using the conventional method and the proposed method. (**a**) Slice at *z* = 0 m using the conventional method; (**b**) Slice at *z* = 0 m using the proposed method; (**c**) Slice at *z* = 0.0075 m using the conventional method; (**d**) Slice at *z* = 0.0075 m using the proposed method; (**e**) Slice at *z* = −0.0075 m using the conventional method; (**f**) Slice at *z* = −0.0075 m using the proposed method.

The experimental imaging results are given in Figure 11, Figure 12, Figure 13, Figure 14 and Figure 15. Figure 11 shows the 3-D imaging results of the scissors with 256 × 256 × 128-point using the conventional method and our method, respectively. In order to have a good view, the 3-D results are displayed with a dynamic range of −15~0 dB. We can see that both methods can reconstruct the basic 3-D shape of the scissors (two semi-circles of handle and a line of blade). However, when compared with the conventional interpolation method, the proposed method behaves better with respect to the contour continuity and clarity. It can also clearly be seen that the results from the conventional method have many noisy artifacts distributed around the scissors, while our method still has no obvious noisy artifacts in the down-sampling rate case. To further compare the imaging performance, we select three slices in each dimension and give the results. Figure 12 shows the slice at *z* = 0 m, where it can be seen that the front view of the scissors reconstructed by the proposed method is better focused than the conventional method, with less clutter and fewer noisy artifacts. Figure 13 is the slice at *y* = 0 m and Figure 14 is the slice at *x* = 0 m. Both slices for our proposed method have better vision effect with lower clutter. More importantly, the results of our method tend to be less noisy than those using the conventional method, which is an important property for real applications. Comparing the reconstructed 3-D images and the slices, we can see that the results of the conventional method have many noisy artifacts randomly distributed around the imaged target, while our method has a much better background.

We also compare some slices at different depths of z in Figure 15 to discuss the geometry information along the depth direction. It is important for concealed target detection and recognition. Slices at *z* = 0 m, *z* = 7.5 mm, *z* = −7.5 mm are presented. Different layers of the scissors are shown. It can be found that the result of our method has better contour and less noise than that of the conventional interpolation method. This scissors are put next to one surface of the paper box and the millimeter waves penetrate this paper surface to reconstruct the scissors. The thickness of the scissors along *z* direction is thin, and the three slices reflect three states of the scissors. The slice at *z* = −7.5 mm reflects the edge of the scissors next to the paper surface and is affected heavily by the paper surface, the slice at *z* = 0 m reflects the main body of the scissors which is affected slightly by the paper surface, and the slice at *z* = 7.5 mm reflects the other edge of the scissors away from the paper surface, whose image intensity is relatively weak. There is a tiny gap in the contour of the scissors, which may be caused by the spatial posture and the scattering characteristics of the target.

In this experiment, the down-sampling measured data are extended to a 256 × 256 × 128 matrix by zero-padding for image reconstruction. The processing times for the conventional method and our method are 32.31 s and 43.77 s, respectively. The interpolation-FFT method is always fast and our method is comparable to some extent. The main advantage of down-sampling measuring is to reduce the acquiring time of the scattered data, which is important for real applications.

Near-field concealed targets imaging impose great demands on the image quality and accuracy since they have a great effect on the latter detection and recognition of concealed targets. The FGG-NUFFT-based 3-D imaging method is efficient and accurate even in a down-sampled case. By using down-sampled data, the measuring time can be reduced, which is important for near-field 3-D imaging.

## 6. Conclusions

In this paper, an accurate algorithm for fast formation of near-field 3-D images via FGG-NUFFT is presented. We tried to introduce FGG-NUFFT into the near-field 3-D imaging area since the imaging procedure also needs to deal with the nonuniform Fourier transforming problem. Simulation and experimental results have shown that the FGG-NUFFT-based method is comparable with the interpolation-FFT method in both accuracy and efficiency when the scattered data are sampled densely enough. We also discussed a case with down-sampled measurements, which is important for real applications of the near-field 3-D imaging. The FGG-NUFFT-based method can have better imaging performance with less clutter and fewer noisy artifacts. This paper is an elementary study of the FGG-NUFFT-based method used in near-field imaging with uniform sampled data with a sufficient sampling rate or down-sampling rate. We will further study the potential of FGG-NUFFT used in compressed sensing imaging or MIMO array imaging in future work.

## Figures and Tables

**Table 1:** Procedure of FGG-NUIFFT.

**Step I: Initialization**
Set the over sampling rate R, the spreading Gaussian parameter $M_{sp}$, and the Gaussian kernel parameter τ. Precompute coefficients $E_{3}(l)=e^{-{\left(\pi l/M_{r}\right)}^{2}/\tau }$ for $0\leq l\leq M_{sp}$ and $E_{4}(u)=E_{4}(M-u)=e^{\tau u^{2}}$ for |k|≤M/2. This step can be precomputed.
**Step II: Convolution for Each Source Point**
find the nearest grid point $\xi =\frac{2\pi m}{M_{r}}$ with $\xi \leq v_{j}$ Compute $E_{1}=e^{-{\left(v_{j}-\xi \right)}^{2}/4\tau }$ $E_{2v}=e^{(v_{j}-\xi )\pi /M_{r}\tau }$ $E_{2v}(l)={E_{2v}}^{l}$ for $-M_{sp}<l\leq M_{sp}$ Convolve the Gaussian spreading function with $f_{i}$ as follows: $$\begin{array}{l} V_{0}=f_{i}\cdot E_{1}\\ \;for\;l_{v}=-M_{sp}+1,M_{sp}\\ \;\;Add\;V_{0}\cdot E_{2v}(l)\;\mathrm{to}\;f_{\tau }(m+l) \end{array}$$
This step costs $O(4M_{sp}\cdot N)$ operations.
**Step III: IFFT and Deconvolution**
Compute IFFT of $f_{\tau }(m)$ to obtain $F_{\tau }(u)$ Deconvolution $F(u)=\sqrt{\frac{\pi }{\tau }}E_{4}(u)F_{\tau }(u)$
This step requires $O(M_{r}log_{2}M_{r})$ operations.

**Table 2:** Position coordinates of scatterers.

Scatterers *i*	*x*-axis (m)	*y*-axis (m)	*z*-axis (m)
1	0	0	0
2	0.1	0.1	0.1
3	0	0.1	0
4	0	0	0.1
5	−0.1	0	0
6	0	−0.1	0
7	0	0	−0.1
